# Quantitative analysis of a Māori and Pacific admission process on first-year health study

**DOI:** 10.1186/s12909-015-0470-7

**Published:** 2015-11-03

**Authors:** Elana Curtis, Erena Wikaire, Yannan Jiang, Louise McMillan, Robert Loto, Airini, Papaarangi Reid

**Affiliations:** 1 Te Kupenga Hauora Māori, Faculty of Medical and Health Sciences, University of Auckland, Private Bag 92015, Auckland, New Zealand; 2 Department of Statistics, Faculty of Science, University of Auckland, Private Bag 92015, Auckland, New Zealand; 3 Faculty of Human, Social and Educational Development, Thompson Rivers University, Thompson, BC Canada

**Keywords:** Admission, Selection, Indigenous, Ethnic minority, Health professional, Higher education, Widening participation, Workforce development, Māori, Pacific

## Abstract

**Background:**

Universities should provide flexible and inclusive selection and admission policies to increase equity in access *and* outcomes for indigenous and ethnic minority students. This study investigates an equity-targeted admissions process, involving a Multiple Mini Interview and objective testing, advising Māori and Pacific students on their *best starting point* for academic success towards a career in medicine, nursing, health sciences and pharmacy.

**Methods:**

All Māori and Pacific Admission Scheme (MAPAS) interviewees enrolled in bridging/foundation or degree-level programmes at the University of Auckland were identified (2009 to 2012). Generalised linear regression models estimated the predicted effects of admission variables (e.g. *MAPAS Maths Test*; *National Certificate in Educational Achievement* (*NCEA) Rank Score; Any 2 Sciences*; *Followed MAPAS Advice)* on first year academic outcomes (i.e. Grade Point Average (GPA) and Passes All Courses) adjusting for *MAPAS interview year*, *gender*, *ancestry* and *school decile*.

**Results:**

368 *First Year Tertiary* (bridging/foundation or degree-level) and 242 *First Year Bachelor* (degree-level only) students were investigated. *NCEA Rank Score* (estimate 0.26, CI: 0.18-0.34, *p*< 0.0001); *MAPAS Advice Followed* (1.26, CI: 0.18-1.34, *p* = 0.0002); *Exposure to Any 2 Sciences* (0.651, CI: 0.15-1.15, *p* = 0.012); and *MAPAS Mathematics Test* (0.14, CI: 0.02-0.26, *p* = 0.0186) variables were strongly associated with an increase in *First Year Tertiary GPA*. The odds of passing all courses in *First Year Tertiary* study was 5.4 times higher for students who *Followed MAPAS Advice* (CI: 2.35-12.39; *p*< 0.0001) and 2.3 times higher with *Exposure to Any Two Sciences* (CI: 1.15-4.60; *p* = 0.0186). *First Year Bachelor* students who *Followed MAPAS Advice* had an average GPA that was 1.1 points higher for all eight (CI: 0.45-1.73; *p* = 0.0009) and Core 4 courses (CI: 0.60-2.04; *p* = 0.0004).

**Conclusions:**

The MAPAS admissions process was strongly associated with positive academic outcomes in the first year of tertiary study. Universities should invest in a comprehensive admissions process that includes alternative entry pathways for indigenous and ethnic minority applicants.

## Background

Worldwide, tertiary institutions are attempting to widen participation to historically underserved populations including indigenous and ethnic minority students [1] Often driven by social inclusion and social accountability policies, universities have devised a number of strategies to increase diversity. Within an indigenous and ethnic minority health workforce context, a pipeline approach is recommended to address well-known barriers to accessing and succeeding in university-level studies. A pipeline approach often includes early exposure interventions aimed at raising aspirations and academic preparation for a career in health [2–4]; addressing educational disadvantage via the provision of bridging/foundation programmes [5, 6] and improving student performance by providing comprehensive support programmes [7–9]. Given the highly competitive context of health professional programme selection, it is also recommended that universities provide more flexible and inclusive selection and admission policies for students from underserved populations [1, 10].

Universities have a choice of selection tools that can be used to inform student admission including prior academic performance, interview scores and results from aptitude tests. Both cognitive and non-cognitive tools are used by universities when selecting students; however it is arguable that prior academic performance remains a dominant tool for medical selection in many universities [11]. Given this reality, indigenous and ethnic minority students are required to aim to achieve a high level of academic performance within the pathways used for future selection into medical or health professional programmes of study [12]. Unfortunately, students from underserved populations are less likely to receive access to science-rich subjects and are more likely to leave high school with lower qualifications than their peers [5, 10, 13]. Providing an admissions process that can determine whether indigenous and ethnic minority applicants are academically (and socially) ready to achieve success in pre-medical degree pathways *and* the provision of alternative entry pathways is recommended for tertiary institutions committed to widening participation [14, 15].

An extensive body of research identifies the tertiary conditions and factors that impact on academic success within the first year of study at university [16–20]. Indicators of prior academic performance such as: secondary school grade point averages [21]; secondary school factors including markers of socio-geographic status (e.g. school decile) [22]; and student characteristics (e.g. autonomy, confidence, motivation, control) [17, 23] have been identified as important factors impacting on academic performance in the first year of study. In addition, factors associated with the environment of the tertiary institution also impact on student engagement; such factors include: opportunities for teachers and students to engage with each other [18]; levels of institutional support to provide environments conducive to learning [20]; and the provision of academic, social and personal support [16].

To date, few studies have explored the effect of equity-targeted admission processes on the academic performance of indigenous and ethnic minority students in their first year of tertiary study. As a result, tertiary institutions have little empirical evidence to understand the effect of equity-targeted selection processes and whether such initiatives are likely to support a widening participation agenda.

This article explores the predictive effect of admission variables associated with an equity-targeted admission process on academic outcomes for Māori (the indigenous peoples of Aotearoa New Zealand) and Pacific (a heterogeneous composite of peoples with Pacific nation ancestry born and/or living in New Zealand) applicants applying under the Māori and Pacific Admission Scheme (MAPAS) to the Faculty of Medical and Health Sciences (FMHS) at the University of Auckland (UoA).

## Methods

### FMHS entry pathways

Admission into FMHS health professional programmes is generally via direct entry into *First Year Bachelor* level undergraduate study for those applicants who meet the necessary entry requirements [24]. The FMHS also offers a one-year, MAPAS-specific bridging/foundation programme, the *Certificate in Health Sciences* (CertHSc) through which Māori and Pacific students who achieve a CertHSc GPA above B+ can gain alternative entry into *First Year Bachelor* undergraduate study. Hence, Māori and Pacific *First Year Tertiary* students within FMHS could either be enrolled in the CertHSc bridging foundation programme, or, the first year of bachelor level study (Table 1). The first year of bachelor level study also acts as a ‘pre-medical’ year prior to admission into the FMHS Medical programme in year 2. Table 1 provides definitions of the Certificate in Health Sciences, First Year Tertiary, and First Year Bachelor terms used within this study (Table 1).

**Table 1:** Definition of terms used within the FMHS context

Term	Definition
Certificate in Health Sciences	A 1-year bridging foundation level programme for Māori and Pacific students that provides an alternative entry pathway to the first year of bachelor degree level undergraduate FMHS health programmes
First Year Tertiary	The first year in which a student enrols in a form of study provided by the tertiary institution (e.g. Certificate in Health Sciences or First Year Bachelor)
First Year Bachelor	The first year in which a student enrols in a form of tertiary study at the level of a bachelor degree qualification

### Māori and Pacific Admission Scheme (MAPAS)

MAPAS operates an equity-targeted admissions process for applicants with indigenous Māori and Pacific ancestry. The process aims to gather a broad range of information about Māori and Pacific applicant preparation for tertiary health study. The December interview process involves a Multiple Mini Interview (MMI), an English test and a mathematics test.

The MMI is an alternative form of admission interview that aims to reduce interviewer bias by consisting of a number of short interview stations with multiple interviewers. The MMI has been shown to be reliable, acceptable and feasible in a variety of tertiary health study contexts [25]. In building on the original pilot of the MMI [26], other studies have taken advantage of the intended benefit of the flexibility of station development in their own contexts [27, 28]. Whilst the original authors aimed to assess suitability of applicants as health professionals, the MAPAS MMI aims to assess Māori and Pacific applicant preparation for and potential to succeed in FMHS programmes. In the MAPAS context, the MMI has been redeveloped to include four 8-min stations assessing career aspirations; academic preparation; family support and student information. The MAPAS mathematics and English testing are used in addition to the MAPAS MMI to objectively assess academic numeracy and literacy skills. Using MMI and testing information, two assessments are made about: 1) potential to succeed within the CertHSc, and 2) potential to succeed within the Bachelor of: Health Sciences; Science (Biomedicine)^1^; Nursing; or Pharmacy. Potential to succeed is assessed as: *pass, borderline or fail* (objective testing*)* for the English and mathematics testing *and few, some, or major concerns* (subjective testing) for each MMI station. A MAPAS Recommendations Team reviews the combination of results and provides a provisional MAPAS recommendation (advice regarding the applicant’s recommended *best starting point* given their intended health career) for applicants (and families) on the day of their interview. Recommended starting points are reflected within three categories: (1) *Bachelor* i.e. start at degree-level; (2) *CertHSc* i.e. start at bridging/foundation; or (3) *Not FMHS* i.e. start in a pathway not provided by FMHS (likely to need further academic preparation not offered by the FMHS). Following the release of secondary school results in January, all information is re-reviewed and a final MAPAS recommendation is provided. MAPAS recommendations are not binding if an applicant has met guaranteed entry criteria for any FMHS programme. In this context, the applicant can choose to follow MAPAS advice (or not)^2^.

### Methodology

This study used a Kaupapa Māori Research (KMR) approach, broadly defined and responsive to Pacific research methodologies [29, 30]. This approach recognises that issues associated with power, privilege and agency within society are hypothesised to act similarly on both Māori and Pacific students [31, 32]. In this instance KMR aims to: ensure research outputs are positive for Māori and Pacific students; explicitly challenge ‘victim blame’ or ‘cultural deficit’ analyses that may blame Māori or Pacific students for educational failure; and provide a structural analysis to promote institutional change targeting Māori and Pacific student success [14, 33]. This research was led by senior Māori and Pacific researchers with input from a FMHS advisory group.

### Study design

The predictive effect of MAPAS admission process variables on academic outcomes in the first year of tertiary study was explored. Applicant data were obtained from the MAPAS admissions database and the university’s centralised student data management system for all MAPAS interviewees (2008 – 2011) who subsequently enrolled in relevant tertiary health programmes (2009 – 2012) within the FMHS at the UoA. Approval to complete this research was granted by the University of Auckland Human Participant Ethics Committee (Ref 8110). As per ethics protocols, written informed consent was not required for this research project due to the use of secondary administrative data sources. All secondary data obtained from these datasets were de-identified by an independent research member with no student contact or teaching responsibilities and data analysis occurred via a coding system. Two student cohorts are identified: *First Year Tertiary Students* i.e. students enrolled in either the CertHSc *or* the first year of a bachelor programme in the year following their MAPAS interview; and *First Year Bachelor Students* i.e. students enrolled in a bachelor programme in either the first or second year following their MAPAS interview (may include CertHSc graduates).

### Variables

Demographic variables include: *Year of Admission* (2009–2012); *Gender* (Female, Male); *Ancestry* (Māori, Pacific, Both) and *School Decile* (High, Medium and Low). Secondary schools with a mid-low decile rating have been linked to higher levels of deprivation associated with reduced access to, and outcomes from, tertiary education [34] (Table 2).

**Table 2:** Descriptive summary of first year tertiary and first year bachelor student demographic and outcome variables

Descriptive summary variables	First year tertiary students		First year bachelor students	
	2009 – 2012 (*n* = 368)		2009 – 2012 (*n* = 242)	
*Continuous variables*	*Mean*	*± SD*	*Mean*	*± SD*
Age (Years ± SD)	19.2	4.2	19.0	3.9
*Categorical variables*	*n*	*%*	*n*	*%*
Year of admission				
2009	70	19	26	11
2010	95	26	69	29
2011	108	29	79	32
2012	95	26	68	28
Gender				
Female	248	67	160	66
Male	120	33	82	34
Ancestry				
Māori	137	37	89	37
Pacific	210	57	138	57
Both Māori and Pacific	21	6	15	6
School Decile				
High (8–10)	82	24	59	26
Medium (4–7)	144	41	98	43
Low (1–3)	123	35	71	31
*Missing*	*19*	-	*14*	-
*Continuous variables*	*Mean*	*± SD*	*Mean*	*± SD*
Grade Point Average (GPA)				
Eight Courses	4.3	2.0	4.1	2.1
Core 4 Courses	–	–	3.8	2.4
*Categorical variables*	*n*	*%*	*n*	*%*
Passes All Eight Courses				
Yes	276	75	145	60
No	92	25	97	40
Passes All Core 4 Courses				
Yes	–	–	154	64
No	–	–	88	36

Admission predictor variables include: *MAPAS Testing* results (%); *MMI Station* results (Some or Major Concerns (SMC) versus Few Concerns (FC)); *Provisional December Recommendation* (CertHSc, Bachelor, Not FMHS); secondary school results including New Zealand’s *NCEA Rank Score*^3^ (out of 320); Level 3 NCEA *Subject Credits* (number of credits achieved in English, biology, chemistry, physics, mathematics); *Exposure to Any 2 Sciences* of senior biology, chemistry or physics (yes, no)^4^; *Followed MAPAS Advice* (yes, no); and *Final January Recommendation* made in January (CertHSc, Bachelor, Not FMHS).

Academic outcome variables include: *Grade Point Average (GPA) Eight Courses,* 0*–*9 (i.e. GPA achieved across a total of eight courses over the year); *GPA Core 4 Courses,* 0*–*9 (i.e. GPA achieved across four core courses^5^ taken in the first year of bachelor study that are specifically assessed for selection into second year medicine at the UoA); *Passes All Courses,* yes/no (i.e. across total of eight courses); *Passes All Core 4 Courses,* yes/no (i.e. across the four core courses).

### Statistical analysis

All downloaded data were recorded in Microsoft Office Excel spread sheets. Statistical analyses were performed using SAS version 9.3 (SAS Institute, Cary, NC, USA). Continuous variables were presented as mean and standard deviation (SD); categorical variables as frequencies (n) and percentages (%) (Tables 2 and 3). Generalised linear and logistic regression models were used to estimate the predicted effects of individual admission variables on academic outcomes (i.e. GPA and Passes All); adjusting for pre-defined demographic variables (i.e. MAPAS interview year, gender, ancestry and school decile) (Tables 4, 5, 6 and 7). Admission variables that showed significant single predictive effect (i.e. MAPAS Maths Test, NCEA Rank Score, Any 2 Sciences and Followed MAPAS Advice) were included in the multiple regression analyses to determine their joint effects on the academic outcomes of interest (Tables 8 and 9). All statistical tests were two-sided at 5 % significance level.

**Table 3:** Descriptive summary of first year tertiary and first year bachelor student predictor variables

Predictors	First year tertiary students		First year bachelor students	
	2009 – 2012 (*n* = 368)		2009 – 2012 (*n* = 242)	
*Continuous variables*	*n*	*Mean ± SD*	*n*	*Mean ± SD*
MAPAS testing				
Mathematics test	241	79.0 *±* 18.3	241	80.4 *±* 18.3
English test	241	68.4 *±* 13.6	241	70.6 *±* 12.8
*Categorical variables*	*n*	*%*	*n*	*%*
CertHSc MMI				
Whānau Support				
FC^d^	305	83	208	86
SMC	63	17	34	14
Academic Preparation				
FC	306	83	210	87
SMC	62	17	32	13
Career Aspirations				
FC	296	80	202	84
SMC	72	20	40	16
Student Information				
FC	295	80	206	85
SMC	73	20	36	15
Bachelor MMI				
Whānau Support				
FC	250	68	178	74
SMC	118	32	64	26
Academic Preparation				
FC	207	56	157	65
SMC	161	44	85	35
Career Aspirations				
FC	296	80	125	52
SMC	72	20	117	48
Student Information (missing = 1)				
FC	205	56	146	61
SMC	162	44	95	39
December Recommendation (Provisional)				
CertHSc	197	55	112	48
Bachelor	131	37	109	47
Not FMHS	28	8	12	5
*Missing*	*12*	–	*9*	–
*Continuous variables*	*n*	*Mean ± SD*	*n*	*Mean ± SD*
School Results (NCEA)^a^				
Rank Score	291	190.5 ± 51.3	194	201.8 *±* 52.7
L3 English^b^	225	16.7 *±* 5.8	150	17.7 *±* 5.6
L3 Biology	260	15.4 *±* 6.1	172	16.8 *±* 5.9
L3 Chemistry	233	14.6 *±* 7.1	165	15.7 *±* 7.0
L3 Physics	132	15.3 *±* 7.8	99	16.6 *±* 7.8
L3 Maths	266	24.2 *±* 13.7	177	26.3 *±* 14.5
*Categorical variables*				
Any 2 sciences (NCEA, CIE, IB)^c^	*n*	*%*	*n*	*%*
Yes	244	66	171	85
No	55	15	31	15
*AA/no school results*	*69*	–	*40*	–
Followed advice				
Yes	315	88.0	196	83
No	43	12.0	39	17
*Missing*	*10*	–	*7*	
January Recommendation (Final)				
CertHSc	256	71.5	137	58
Bachelor	95	26	91	39
Not FMHS	7	2	7	3
*Missing*	*10*	–	*7*	–

^a^Rank Score and L3 subject results analysis was completed for applicants who completed the National Certificate in Educational Achievement (*NCEA*) only. Excludes Cambridge International Exam (*CIE*), International Baccalaureate (*IB*), International students, alternative admission applicants and missing data

^b^L3 subject missing data includes those NCEA applicants who did not enrol in that particular subject

^c^Any 2 sciences was calculated for all applicants who had available subject results for any two of the three applied science subjects (Physics, Biology, and Chemistry). N for any 2 sciences differs from Rank Score as it does not exclude CIE, IB, International, or alternative admission students

^d^*FC* Few concerns, *SMC* Some or major concerns

**Table 4:** Univariate regression analysis results – GPA eight courses

Predictors	First year tertiary students		First year bachelor students	
	2009 – 2012 (*n* = 368)		2009 – 2012 (*n* = 242)	
	Mean estimate (95 % CI)	*P* value	Mean estimate (95 % CI)	*P* value
GPA Eight Courses				
Any 2 sciences (NCEA, CIE, IB)**				
No	0.00		0.00	
Yes	0.971 (0.44, 1.50)	0.0004*	0.912 (0.17, 1.65)	0.0169
Followed advice				
No	0.00		0.00	
Yes	0.78 (0.18, 1.38)	0.0109*	0.84 (0.17, 1.51)	0.0147*
CertHSc MMI				
Whānau Support				
FC^a^	0.00		0.00	
SC	0.14 (−0.43, 0.71)	0.6201	0.66 (−0.12, 1.44)	0.0972
MC	−1.5 (−2.98, −0.02)	0.0475	−1.41 (−4.24, 1.42)	0.3290
Academic Preparation				
FC	0.00		0.00	
SC	−0.27 (−0.87, 0.33)	0.3799	−0.29 (−1.18, 0.60)	0.5254
MC	0.56 (−0.50, 1.62)	0.2989	0.93 (−0.66, 2.52)	0.2531
Career Aspirations				
FC	0.00		0.00	
SC	−0.83 (−1.39, −0.28)	0.0035*	−1.10 (−1.90, −0.29)	0.0081*
MC	−0.28 (−1.56, 1.00)	0.6676	1.12 (−0.53, 2.77)	0.1833
Student Information				
FC	0.00		0.00	
SC	1.28 (0.72, 1.84)	0.3100	−0.47 (−1.29, 0.34)	0.2572
MC	−0.29 (−1.60, 1.02)	0.0559	2.06 (−0.26, 4.37)	0.0834
Bachelor MMI				
Whānau Support			0.00	
FC	0.00		0.03 (−0.65, 0.71)	
SC	−0.07 (−0.56, 0.42)	0.2503	0.59 (−0.56, 1.74)	0.346
MC	−0.38 (−1.22, 0.46)	0.4301		0.586
Academic Preparation				
FC	0.00		0.00	
SC	−0.08 (−0.59, 0.43)	0.2601	−0.04 (−0.71, 0.64)	0.345
MC	−0.15 (−0.76, 0.46)	0.3112	0.05 (−0.85, 0.96)	0.463
Career Aspirations				
FC	0.00		0.00	
SC	−0.73 (−1.18, −0.28)	0.2315	−0.77 (−1.37, −0.17)	0.307
MC	−0.79 (−1.40, −0.19)	0.3076	−0.74 (−1.56, 0.08)	0.419
Student Information				
FC	0.00		0.00	
SC	−0.04 (−0.50, 0.41)	0.2344	−0.13 (−0.73, 0.47)	0.306
MC	−0.25 (−0.95, 0.45)	0.3564	−1.23 (−2.20, −0.25)	0.497
*Continuous variables*				
School Results (NCEA)*				
Rank Score (per 20 pt increase)	0.26 (0.18, 0.34)	<0.0001*	0.36 (0.26, 0.44)	<0.0001*
L3 English^	−0.005 (−0.09, 0.08)	0.912	−0.006 (−0.09, 0.08)	0.9014
L3 Biology	0.051 (−0.03, 0.14)	0.249	0.034 (−0.06, 0.13)	0.4711
L3 Chemistry	0.001 (−0.08, 0.08)	0.987	−0.044 (−0.13, 0.04)	0.3039
L3 Physics	0.091 (0.03, 0.15)	0.004*	0.06 (−0.004, 0.13)	0.0708
L3 Maths	0.008 (−0.03, 0.05)	0.664	0.036 (−0.01, 0.08)	0.0964
MAPAS Maths test (per 10 % increase)	0.23 (0.11, 0.35)	0.0002*	0.18 (0.03, 0.34)	0.0233*
MAPAS English test(per 10 % increase)	0.09 (−0.09, 0.26)	0.324	0.05 (−0.19, 0.29)	0.6834

^ L3 subject missing data includes those NCEA applicants who did not enrol in that particular subject

*Adjusted for MAPAS interview year, gender, ancestry and school decile. For GPA (a continuous outcome variable), its mean change associated with the change in alinear predictor was estimated with 95 % confidence interval. For a continuous predictor variable, this gave the difference in means with either 20 point (NCEA Rank Score) or 10 % (MAPAS Maths percentage mark) increase in the predictor. For a categorical predictor, this gave the difference in means between the current and reference categories (i.e. yes vs. no). The null hypothesis was that there was no change in the mean response (i.e. Δ = 0)

**NCEA = National Certificate in Educational Achievement, CIE = Cambridge International Exam, IB = International Baccalaureate

^a^*FC* Few concerns, *SMC* Some or major concerns

**Table 5:** Univariate regression analysis results – GPA core 4 courses

Predictors	First year tertiary students		First year bachelor students	
	2009 – 2012 (*n* = 368)		2009 – 2012 (*n* = 242)	
	Mean estimate (95 % CI)	*P* value	Mean estimate (95 % CI)	*P* value
GPA Core 4 Courses				
Any 2 sciences (NCEA, CIE, IB)**				
No			0.00	
Yes			1.12 (0.30, 1.94)	0.0082*
Followed advice				
No			0.00	
Yes			1.10 (0.36, 1.84)	0.0040
CertHSc MMI				
Whānau Support				
FC^a^			0.00	
SC			0.75 (−0.15, 1.61)	0.0909
MC			−0.34 (−3.48, 2.79)	0.8300
Academic Preparation				
FC			0.00	
SC			−0.32 (−1.30, 0.67)	0.5259
MC			0.91 (−0.86, 2.67)	0.3145
Career Aspirations				
FC			0.00	
SC			−1.37 (−2.26, −0.48)	0.0029*
MC			1.21 (−0.62, 3.04)	0.1961
Student Information				
FC			0.00	
SC			−0.66 (−1.57, 0.24)	0.1532
MC			2.60 (0.02, 5.17)	0.0490
Bachelor MMI				
Whānau Support				
FC			0.00	
SC			0.09 (−0.67, 0.85)	0.8159
MC			0.52 (−.076, 1.81)	0.4249
Academic Preparation				
FC			0.00	
SC			−0.10 (−0.86, 0.65)	0.7887
MC			−0.10 (−1.12, 0.92)	0.8484
Career Aspirations				
FC			0.00	
SC			−0.77 (−1.45, −0.10)	0.0256
MC			−0.74 (−1.66, 0.18)	0.1179
Student Information				
FC			0.00	
SC			−0.22 (−0.89, 0.46)	0.5299
MC			−1.19 (−2.29, −0.10)	0.0331
*Continuous variables*				
School Results (NCEA)*				
Rank Score			0.34 (0.24, 0.46)	<0.0001*
L3 English^			−0.03 (−0.13, 0.06)	0.5145
L3 Biology			0.04 (−0.06, 0.14)	0.4349
L3 Chemistry			−0.05 (−0.14, 0.04)	0.2837
L3 Physics			0.07 (0.001, 0.14)	0.0528
L3 Maths			0.04 (−0.003, 0.09)	0.0734
MAPAS Maths test (per 10 % increase)			0.26 (0.09, 0.44)	0.0039*
MAPAS English test(per 10 % increase)			0.03 (−0.24, 0.29)	0.8523

^ L3 subject missing data includes those NCEA applicants who did not enrol in that particular subject

^a^ *FC* Few concerns

*Adjusted for MAPAS interview year, gender, ancestry and school decile. For GPA (a continuous outcome variable), its mean change associated with the change in alinear predictor was estimated with 95 % confidence interval. For a continuous predictor variable, this gave the difference in means with either 20 point (NCEA Rank Score) or 10 % (MAPAS Maths percentage mark) increase in the predictor. For a categorical predictor, this gave the difference in means between the current and reference categories (i.e. yes vs. no). The null hypothesis was that there was no change in the mean response (i.e. Δ = 0)

**NCEA = National Certificate in Educational Achievement, CIE = Cambridge International Exam, IB = International Baccalaureate

**Table 6:** Univariate regression analysis results – passes all eight courses

Predictors	First year tertiary students		First year bachelor students	
	2009 – 2012 (*n* = 368)		2009 – 2012 (*n* = 242)	
	Odds ratio (95 % CI)	Overall *P* value	Odds ratio (95 % CI)	Overall *P* value
Passes All Eight Courses				
Any 2 sciences (NCEA, CIE, IB)**				
No	1.00		1.00	
Yes	2.52 (1.32, 4.83)	0.005*	1.90 (0.87, 4.15)	0.106
Followed advice				
No	1.00		1.00	
Yes	3.30 (1.67, 6.52)	0.001*	1.97 (0.98, 3.98)	0.058
CertHSc MMI				
Whānau Support				
FC^b^	1.00		1.00	
SC	1.21 (0.59, 2.49)		1.60 (0.68, 3.72)	
MC	0.19 (0.03, 1.07)	0.130	0.64 (0.04, 11.26)	0.520
Academic Preparation				
FC	1.00		1.00	
SC	0.81 (0.39, 1.68)		0.89 (0.35, 2.27)	
MC	1.67 (0.39, 7.18)	0.642	1.52 (0.28, 8.29)	0.850
Career Aspirations				
FC	1.00		1.00	
SC	0.47 (0.24, 0.91)		0.38 (0.14, 0.80)	
MC	0.47 (0.10, 2.13)	0.061	1.32 (0.22, 7.87)	0.042*
Student Information				
FC	1.00		1.00	
SC	1.26 (0.61, 2.59)		1.15 (0.47, 2.83)	
MC	4.11 (0.46, 36.87)	0.395	>999.999^a^	0.951
Bachelor MMI				
Whānau Support				
FC	1.00		1.00	
SC	0.78 (0.43, 1.41)		0.79 (0.39, 1.63)	
MC	0.79 (0.29, 2.14)	0.686	1.65 (0.46, 5.95)	0.541
Academic Preparation				
FC	1.00		1.00	
SC	1.58 (0.82, 3.05)		0.86 (0.42, 1.78)	
MC	1.02 (0.48, 2.16)	0.326	0.90 (0.35, 2.33)	0.920
Career Aspirations				
FC	1.00		1.00	
SC	0.88 (0.49, 1.58)		0.57 (0.30, 1.08)	
MC	0.77 (0.36, 1.64)	0.791	0.74 (0.31, 1.77)	0.228
Student Information				
FC	1.00		1.00	
SC	0.82 (0.46, 1.47)		0.77 (0.41, 1.47)	
MC	0.97 (0.41, 2.31)	0.799	0.50 (0.18, 1.38)	0.375
*Continuous variables*				
School Results (NCEA)*				
Rank Score (per 20 pt increase)	1.08 (0.96, 1.20)	0.178	1.35 (1.17, 1.54)	<0.0001*
L3 English^	1.003 (0.87, 1.16)	0.971	0.95 (0.81, 1.10)	0.485
L3 Biology	1.04 (0.90, 1.20)	0.575	1.19 (0.99, 1.43)	0.060
L3 Chemistry	0.96 (0.84, 1.11)	0.602	0.78 (0.65, 0.94)	0.010*
L3 Physics	1.15 (1.01, 1.31)	0.039*	1.10 (0.95, 1.28)	0.196
L3 Maths	1.06 (0.98, 1.15)	0.167	1.23 (1.06, 1.44)	0.008*
MAPAS Maths test (per 10 % increase)	1.17 (1.01, 1.36)	0.033*	1.19 (1.02, 1.42)	0.032*
MAPAS English test(per 10 % increase)	0.94 (0.75, 1.17)	0.595	0.84 (0.65, 1.09)	0.202

*Adjusted for MAPAS interview year, gender, ancestry and school decile. For Passes All Courses (a binary outcome variable), the odds ratio (OR) associated with the change in a linear predictor was estimated with 95 % confidence interval. For a continuous predictor, this indicated the difference in ratio of two odds with either 20 point (NCEA Rank Score) or 10 % (MAPAS Maths test) increase in the predictor, relative to the odds with no increase. For a categorical predictor, this indicated the difference in odds between the current and reference categories (e.g. the odds of Passes All Courses with exposure to Any 2 Sciences, relative to the odds of not having exposure to Any 2 Sciences). The null hypothesis was that there was no change in the odds (i.e. OR = 1)

**NCEA = National Certificate in Educational Achievement, CIE = Cambridge International Exam, IB = International Baccalaureate

^a^ Insufficient data available for analysis

^b^ *FC* Few concerns, *SMC* Some or major concerns

^ L3 subject missing data includes those NCEA applicants who did not enrol in that particular subject

**Table 7:** Univariate regression analysis results: passes all core 4 courses

Predictors	First year tertiary students		First year bachelor students	
	2009 – 2012 (*n* = 368)		2009 – 2012 (*n* = 242)	
	Odds ratio (95 % CI)	Overall *P* value	Odds ratio (95 % CI)	Overall *P* value
Passes All Core 4 Courses				
Any 2 sciences (NCEA, CIE, IB)**				
No			1.00	
Yes			2.57 (1.16, 5.68)	0.020*
Followed advice				
No			1.00	
Yes			1.83 (0.90, 3.71)	0.095
CertHSc MMI				
Whānau Support				
FC^a^			1.00	
SC			1.51 (0.64, 3.57)	
MC			0.54 (0.03, 9.69)	0.581
Academic Preparation				
FC			1.00	
SC			0.79 (0.31, 2.03)	
MC			1.35 (0.25, 7.38)	0.818
Career Aspirations				
FC			1.00	
SC			0.36 (0.15, 0.84)	
MC			1.21 (0.19, 7.52)	0.059
Student Information				
FC			1.00	
SC			1.03 (0.42, 2.54)	
MC			>999.999^†^	0.998
Bachelor MMI				
Whānau Support				
FC			1.00	
SC			0.70 (0.34, 1.46)	
MC			1.51 (0.41, 5.53)	0.453
Academic Preparation				
FC			1.00	
SC			0.76 (0.36, 1.59)	
MC			1.01 (0.38, 2.66)	0.737
Career Aspirations				
FC			1.00	
SC			0.54 (0.28, 1.05)	
MC			0.60 (0.25, 1.46)	0.175
Student Information				
FC			1.00	
SC			0.69 (0.36, 1.32)	
MC			0.44 (0.16, 1.24)	0.240
*Continuous variables*				
School Results (NCEA)*				
Rank Score			1.37 (1.20, 1.57)	<0.0001*
L3 English^			1.01 (0.83, 1.23)	0.921
L3 Biology			1.20 (0.95, 1.51)	0.134
L3 Chemistry			0.85 (0.70, 1.03)	0.089
L3 Physics			1.10 (0.94, 1.29)	0.213
L3 Maths			1.27 (1.04, 1.54)	0.017*
MAPAS Maths test (per 10 % increase)			1.21 (1.02, 1.42)	0.029*
MAPAS English test(per 10 % increase)			0.99 (0.96, 1.01)	0.283

*Adjusted for MAPAS interview year, gender, ancestry and school decile. For Passes All Courses (a binary outcome variable), the odds ratio (OR) associated with the change in a linear predictor was estimated with 95 % confidence interval. For a continuous predictor, this indicated the difference in ratio of two odds with either 20 point (NCEA Rank Score) or 10 % (MAPAS Maths test) increase in the predictor, relative to the odds with no increase. For a categorical predictor, this indicated the difference in odds between the current and reference categories (e.g. the odds of Passes All Courses with exposure to Any 2 Sciences, relative to the odds of not having exposure to Any 2 Sciences). The null hypothesis was that there was no change in the odds (i.e. OR = 1)

**NCEA = National Certificate in Educational Achievement, CIE = Cambridge International Exam, IB = International Baccalaureate

^a^*FC* Few concerns, *SMC* Some or major concerns

^†^Insufficient data available for analysis

**Table 8:** Multiple regression analysis results – linear regression^a^

Multivariate analysis results	First year tertiary students		First year bachelor students	
	2009 – 2012 (*n* = 368)		2009 – 2012 (*n* = 242)	
	Mean estimate (95 % CI)	*P* value	Mean estimate (95 % CI)	*P* value
GPA Eight Courses				
NCEA Rank Score (per 20 point increase)	0.26 (0.18, 0.34)	<0.0001	0.40 (0.30, 0.50)	<0.0001
Followed MAPAS advice				
No	0.00		0.00	
Yes	1.17 (0.57, 1.78)	0.0002	1.09 (0.45, 1.73)	0.0009
Any 2 sciences				
No	0.00		0.00	
Yes	0.65 (0.15, 1.15)	0.0116	0.39 (−0.29, 1.08)	0.2603
MAPAS Maths test (per 10 % increase)	0.14 (0.02, 0.26)	0.0186	0.08 (−0.07, 0.22)	0.2885
GPA Core 4 Courses				
NCEA Rank Score (per 20 point increase)	-	-	0.38 (0.26, 0.50)	<0.0001
Followed MAPAS advice	-	-		
No			0.00	
Yes	-	-	1.14 (0.60, 2.04)	0.0004
Any 2 sciences	-	-		
No			0.00	
Yes	-	-	0.64 (−0.13, 1.41)	0.1027
MAPAS Maths test (per 10 % increase)	-	-	0.15 (−0.02, 0.31)	0.0765

^a^ Adjusted for MAPAS interview year, gender, ancestry and school decile. For GPA (a continuous outcome variable), its mean change associated with the change in a linear predictor was estimated with 95 % confidence interval. For a continuous predictor variable, this gave the difference in means with either 20 point (NCEA Rank Score) or 10 % (MAPAS Maths percentage mark) increase in the predictor. For a categorical predictor, this gave the difference in means between the current and reference categories (i.e. yes vs. no). The null hypothesis was that there was no change in the mean response (i.e. Δ = 0)

**Table 9:** Multiple regression analysis results – logistic regression^a^

Multivariate analysis results	First year tertiary students		First year bachelor students	
	2009 – 2012 (*n* = 368)		2009 – 2012 (*n* = 242)	
	Odds ratio (95 % CI)	*P* value	Odds ratio (95 % CI)	*P* value
Passes All Eight Courses				
NCEA Rank Score (per 20 point increase)	1.10 (0.98, 1.27)	0.112	1.46 (1.24, 1.74)	<0.0001
Followed MAPAS advice				
No	1.00		1.00	
Yes	5.40 (2.36, 12.39)	<0.0001	3.34 (1.45, 7.69)	0.005
Any 2 sciences				
No	1.00		1.00	
Yes	2.30 (1.15, 4.61)	0.019	1.36 (0.55, 3.33)	0.504
MAPAS Maths test (per 10 % increase)	1.13 (0.95, 1.33)	0.179	1.08 (0.90, 1.32)	0.392
Passes All Core 4 Courses				
NCEA Rank Score (per 20 point increase)	–	–	1.48 (1.24, 1.74)	<0.0001
Followed MAPAS advice	–	–		
No			1.00	
Yes	–	–	3.27 (1.39, 7.69)	0.0067
Any 2 sciences	–	–		
No			1.00	
Yes	–	–	1.95 (0.78, 4.84)	0.1513
MAPAS Maths test (per 10 % increase)	–	–	1.10 (0.91, 1.34)	0.3156

^a^Adjusted for MAPAS interview year, gender, ancestry and school decile. For Passes All Courses (a binary outcome variable), the odds ratio (OR) associated with the change in a linear predictor was estimated with 95 % confidence interval. For a continuous predictor, this indicated the difference in ratio of two odds with either 20 point (NCEA Rank Score) or 10 % (MAPAS Maths test) increase in the predictor, relative to the odds with no increase. For a categorical predictor, this indicated the difference in odds between the current and reference categories (e.g. the odds of Passes All Courses with exposure to Any 2 Sciences, relative to the odds of not having exposure to Any 2 Sciences). The null hypothesis was that there was no change in the odds (i.e. OR = 1)

## Results

### Descriptive variables

A total of 368 students were identified in the *First Year Tertiary* cohort. Of these, 37 % were Māori, 57 % Pacific and 6 % had Both Māori and Pacific ancestry. Two thirds were female (67 %), the mean age was 19.2 years (SD 4.2 %) and 70 % or more came from a secondary school with a medium or low school decile (representing more deprived communities). The *First Year Bachelor* cohort had a total of 242 students with a similar demographic profile to *First Year Tertiary* students (Table 2).

### Predictor variables

#### Mathematics and english testing

The *First Year Tertiary* cohort had a mean percentage mark for the mathematics test of 79.0 % (SD 18.3 %) and 68.4 % (SD 13.6 %) for the English test. This represents a *borderline-fail* result for bachelor-level study and a *pass* result for CertHSc-level study as the best starting point of entry across both assessments. *The First Year Bachelor* cohort had a slightly higher mean mark for both the mathematics (80.4 %, SD 18.3 %) and English tests (70.6 %, SD 12.8 %) (Table 3).

#### MMI

Over 80 % of all students from both cohorts were assessed as having *few concerns* for CertHSc-level entry across the four MMI stations. Forty-four percent of all *First Year Tertiary* students were assessed as having *some* or *major concerns* for bachelor-level entry at the Academic Preparation and Student Information MMI stations. For *First Year Bachelor* students, the stations with the highest proportion of *some* or *major concerns* for bachelor-level entry were Career Aspirations (48 %) and Student Information (39 %) (Table 3).

#### School results

The average NCEA rank score (out of a total of 320) was 190.5 (SD 51.3) for *First Year Tertiary* and 201.8 (SD 52.7) for *First Year Bachelor* students. Both averages fall below requirements for guaranteed entry within FMHS (set at a rank score between 210 – 250 depending on the programme). The average number of subject credits for both cohorts were 0.3–3.4 credits below requirements for guaranteed entry (i.e. 16 - 18 subject credits depending on programme) (Table 3). At least two thirds of all students admitted into either the CertHSc or bachelor programmes had taken two or more science subjects in their final year of secondary school (Table 3).

#### MAPAS recommendations

For *First Year Tertiary* students, MAPAS recommended CertHSc to 72 % of all students, followed by Bachelor (26 %) and Not FMHS (2 %). For *First Year Bachelor* students, 58 % were recommended to start at the CertHSc level, followed by 39 % Bachelor and 3 % Not FMHS (Table 3).

#### Followed MAPAS advice

Over 83 % of all students followed MAPAS advice regarding the best starting point for success with only 12 - 17 % of students from each cohort not following their final MAPAS recommendation (Table 3).

### Outcome variables

#### GPA All eight courses and core 4 courses

The average GPA for all eight courses (out of a total of 9) was 4.3 (SD 2.0) for *First Year Tertiary* and 4.1 (SD 2.1) for *First Year Bachelor* students. The average GPA achieved for the Core 4 Courses was 3.8 (SD 2.4) for *First Year Bachelor* students.

#### Passes All eight courses and passes All core 4 courses

Seventy-five percent of *First Year Tertiary* students and 60 % of *First Year Bachelor* students passed all eight courses. Sixty-four percent of *First Year Bachelor* students passed all Core 4 Courses (Table 2).

### Multiple regression analysis

#### First year tertiary - GPA

As shown in Table 8, all predictors had a statistically significant effect on *First Year Tertiary* GPA, with the most significant predictor being NCEA Rank Score, then MAPAS Advice Followed, Any 2 Sciences and MAPAS Mathematics Test results. *First year Tertiary GPA* increased by an average of 0.3 (out of a total 9) for every 20 point increase in NCEA Rank Score (CI: 0.18-0.34; *p* < 0.0001). Students who followed MAPAS advice had on average a GPA that was 1.2 points higher (out of a total 9) than students who did not (CI: 0.57-1.78; *p* = 0.0002).

#### First year tertiary - passes All courses

The odds of passing all eight courses was 5.4 times higher for those students who followed MAPAS advice versus those students who did not (CI: 2.36-12.39; *p* < 0.0001) (Table 8). The odds of passing all eight courses was 2.3 times higher for those students who had exposure to Any 2 Sciences versus those students who did not (CI: 1.15-4.61; *p* = 0.019) (Table 8).

#### First year bachelor - GPA

For every 20 point increase in NCEA Rank Score, the GPA achieved by *First Year Bachelor* students increased by an average of 0.4 for all 8 courses (CI: 0.30-0.50; *p* < 0.0001) and for Core 4 courses (CI: 0.26-0.50; *p* < 0.0001) (Table 7). Students who followed MAPAS advice had on average a GPA that was 1.1 points higher than students who did not follow MAPAS advice for all eight courses (CI: 0.45-1.73; *p* = 0.0009) and Core 4 courses (CI: 0.60-2.04; *p* = 0.0004) (Table 8).

#### First year bachelor - passes All courses

A 20 point increase in NCEA Rank Score increased the odds of passing all first year bachelor courses by a factor of 1.5 (CI: 1.24-1.74; *p* < 0.0001), with similar results for passing all Core 4 courses (Table 8). The odds of passing all first year bachelor courses (CI: 1.45-7.69; *p* = 0.005) and all Core 4 courses (CI: 1.39-7.69; *p* = 0.007) was 3.3 times higher for those students who followed MAPAS advice versus those students who did not (Table 9).

## Discussion

Our findings confirm that the MAPAS admissions process is strongly associated with positive academic outcomes in the first year of tertiary study. Our results reinforce the evidence-base showing a strong association between secondary school performance via NCEA rank score (a marker of the quality of grades achieved) and positive tertiary academic outcomes [35]. The existing literature base has also been extended, given our identification of a strong association between exposure to two or more senior science subjects (a marker of breadth of knowledge) and first year academic outcomes. Similar to other studies, our findings show that the number of credits achieved within NCEA subjects appear to be less strongly correlated with tertiary outcomes [35].

Overall, our findings suggest that there is value in providing a comprehensive admissions process for indigenous and ethnic minority students applying under equity targeted admission programmes. Students admitted into tertiary institutions under targeted admission programmes have been shown to experience peer/educator stigma and ‘everyday racism’. Demonstrating the effectiveness of targeted admission programmes may assist some indigenous and ethnic minority students to override this societal (and potentially internalised) stigma to receive the benefits that targeted admission programmes have to offer.

Increasing the odds of passing all first year courses has relevance for all students. This is important for applicants pursuing medicine as even small increments in first year bachelor GPA, particularly within the Core 4 courses used for medical selection, may have a profound impact on potential selection [12, 19]. A student’s progress towards completion of total point requirements within their degree has been shown to improve student retention and increase the likelihood of degree completion [36]. Aligning MAPAS admission to a comprehensive process focussed on achieving equity in access *and* performance is likely to have contributed to the recent increase in numbers and improved performance observed for Māori and Pacific students within the FMHS [5, 37].

Our data suggests secondary schooling is yet to demonstrate the ability to prepare Māori and Pacific students adequately for tertiary health professional study. Both teaching and subject selection are critical factors. Māori and Pacific students and their families are not to blame for the observed inequities in secondary education. Rather, Māori and Pacific students and their families should receive greater support to navigate NCEA subject selection and ensure that students achieve the right number *and* quality of credits [38]. This is consistent with international evidence showing that indigenous and ethnic minority students are less likely to receive high-quality careers or university advice [38, 39] and in some instances may be actively discouraged from pursuing a health professional career [2].

Based on our findings, it appears that the secondary education sector is failing to ensure that indigenous and ethnic minority students are ‘university-ready’ for health-professional study. Unfortunately, this is not a new issue [5, 14, 40, 41] and nor is it unique to New Zealand [3, 42]. Action by secondary schools and educators to address their own role in the creation and maintenance of ethnic inequities in academic outcomes is recommended [43]. Likewise, tertiary institutions are expected to be part of the solution [44]. Pechenkina & Anderson (2011) call for *“more effective institutional response to the lack of adequate preparation of indigenous students…via greater investment in the pipeline and provision of transitioning programmes*” (p. 5-6). Our findings further support the delivery of bridging/foundation programmes targeting indigenous and ethnic minority students.

### Strengths

This study explores a unique application of the MMI within an equity-targeted context [14, 26]. Although we identified varied associations between individual MMI stations and academic outcomes, we believe that our overall findings support maintaining the MMI within the MAPAS admissions process. This reflects the strong association observed between following MAPAS advice (a predictor variable that is determined by the combined assessment of all results) and higher academic outcomes.

Using both cognitive (e.g. NCEA school results, MAPAS Maths and English test) and non-cognitive (e.g. MMI results) tools for student selection within the total MAPAS admission process supports a widening participation agenda and is consistent with recommendations to use more inclusive selection tools [10, 45-47]. This is particularly important when assessing the potential of alternative admission or older applicants who may possess maturity shown to be positively associated with tertiary programme completion [36, 48].

### Limitations

This study has a number of limitations. The analysis relied on secondary data and is therefore limited by the quality of data sources. However, combining central university and MAPAS datasets has reduced the potential for data misclassification by using verified ancestry and increased the admission variables available for analysis [49, 50]. Our research was limited to first-year outcomes due to resource and time constraints. Ideally, the effect of predictor variables on long-term outcomes across all FMHS programmes should be examined. Comparing academic outcomes across all ethnic groups may also highlight issues of disadvantage *and* privilege [51]. This research is in progress and is drawing on the methods developed within this study. We acknowledge that combining Māori and Pacific data is not ideal from an indigenous rights or Pacific-centric perspective. However, this is consistent with our methodological approach as it maximises statistical power (to aid student success) and supports a structural critique of the effect of ‘society’ *on* ‘ancestry’ [14]. As the quantum of Māori and Pacific data increases, further research should investigate Māori-specific and Pacific-specific predictors of academic success.

## Conclusion

Tertiary institutions committed to widening participation should prioritise the funding and delivery of a comprehensive, flexible and inclusive admissions process that includes alternative entry pathways for indigenous and ethnic minority applicants [10, 52, 53].

### Ethical approval

This project was approved by the University of Auckland Human Participants Ethics Committee, Ref 8110.

## Abbreviations

CertHSc: Certificate in Health Sciences (Hikitia Te Ora)
CIE: Cambridge International Exam
FMHS: Faculty of Medical and Health Sciences
GPA: Grade Point Average
IB: International Baccalaureate
KMR: Kaupapa Māori Research
MAPAS: Māori and Pacific Admission Scheme
NCEA: National Certificate of Educational Achievement
UoA: University of Auckland

## Footnotes

1 Completion of the first year of study within either the Bachelor of Health Sciences or the Bachelor of Science (Biomedicine) programme is required for an undergraduate application to the medical programme at the UoA

2 For additional information, see previous publications 5. Curtis E, Reid P. Indigenous health workforce development: Challenges and successes of the Vision 20: 20 programme. Australian & New Zealand Journal of Surgery. 2013;83(2013):49-54, 13. Curtis E, Reid P, Jones R. Decolonising the Academy: The process of re-presenting indigenous health in tertiary teaching and learning. In: Cram F, Phillips H, Sauni P, Tuagalu C, editors. Māori and Pasifika Higher Education Horizons. Bingley, U.K.: Emerald Group Publishing Limited; 2014. p. 147-66, 14. Curtis, E., Wikaire, E., Jiang, Y., McMillan, L., Loto, R., Airini, & Reid, P. (2015). A tertiary approach to improving equity in health: Quantitative analysis of the Māori and Pacific admission scheme (MAPAS) process, 2008-2012. International Journal for Equity in Health, 14(7). 10.1186/s12939-015-0133-7. or https://www.fmhs.auckland.ac.nz/en/faculty/for/future-undergraduates/maori-and-pacific-admission-scheme.html

3 The National Certificate of Educational Achievement (NCEA) is the major assessment method used in New Zealand secondary schools. The NCEA Rank Score reflects the *best* 80 credits at Level 3 or higher, over a maximum of five approved subjects. It reflects a system of Grade Point Average and is used by the UoA to assist with admission to limited entry programmes 23. Shulruf B, Hattie J, Tumen S. New Zealand’s standard-based assessment for secondary schools (NCEA): implications for policy makers. Asia Pacific Journal of Education. 2010;30(2).

4 Exposure to a minimum of two final year secondary school science subjects is recommended for success within the CertHSc (alongside English and mathematics rich subjects). This variable includes secondary school results from NCEA, International Baccalaureate (IB) and Cambridge International Examinations (CIE).

5 The *Core 4* courses include: CHEM110 (Chemistry of the living world), POPLHLTH 111 (Population Health), MEDSCI 142 (Biology for Biomedicine Science: Organ Systems) and BIOSCI 107 (Biology for Biomedicine Science: Cellular Processes and Development).
